# Towards a Real-Life Understanding of the Altered Functional Behaviour of the Default Mode and Salience Network in Chronic Pain: Are People with Chronic Pain Overthinking the Meaning of Their Pain?

**DOI:** 10.3390/jcm13061645

**Published:** 2024-03-13

**Authors:** Elin Johansson, Huan-Yu Xiong, Andrea Polli, Iris Coppieters, Jo Nijs

**Affiliations:** 1 Pain in Motion Research Group (PAIN), Department of Physiotherapy, Human Physiology and Anatomy, Faculty of Physical Education and Physiotherapy, Vrije Universiteit Brussel, 1090 Brussels, Belgium; elin.johansson@vub.be (E.J.); huanyu.xiong@vub.be (H.-Y.X.); andrea.polli@vub.be (A.P.); iris.coppieters@vub.be (I.C.); 2 Laboratory for Brain-Gut Axis Studies (LaBGAS), Translational Research in Gastrointestinal Disorders (TARGID), Department of Chronic Diseases and Metabolism (CHROMETA), Katholieke Universiteit Leuven, 3000 Leuven, Belgium; 3 Flanders Research Foundation-FWO, 1000 Brussels, Belgium; 4 Department of Public Health and Primary Care, Centre for Environment and Health, Katholieke Universiteit Leuven, 3000 Leuven, Belgium; 5 The Experimental Health Psychology Research Group, Faculty of Psychology and Neuroscience, Maastricht University, 6200 Maastricht, The Netherlands; 6 Chronic Pain Rehabilitation, Department of Physical Medicine and Physiotherapy, University Hospital Brussels, 1090 Brussel, Belgium; 7 Department of Health and Rehabilitation, Unit of Physiotherapy, Institute of Neuroscience and Physiology, Sahlgrenska Academy, University of Gothenburg, 405 30 Gothenburg, Sweden

**Keywords:** chronic pain, default mode network, salience network, ventromedial prefrontal cortex

## Abstract

Chronic pain is a source of substantial physical and psychological suffering, yet a clear understanding of the pathogenesis of chronic pain is lacking. Repeated studies have reported an altered behaviour of the salience network (SN) and default mode network (DMN) in people with chronic pain, and a majority of these studies report an altered behaviour of the dorsal ventromedial prefrontal cortex (vmPFC) within the anterior DMN. In this topical review, we therefore focus specifically on the role of the dorsal vmPFC in chronic pain to provide an updated perspective on the cortical mechanisms of chronic pain. We suggest that increased activity in the dorsal vmPFC may reflect maladaptive overthinking about the meaning of pain for oneself and one’s actions. We also suggest that such overthinking, if negative, may increase the personal “threat” of a given context, as possibly reflected by increased activity in, and functional connectivity to, the anterior insular cortex within the SN.

## 1. Introduction

**Figure 1:**
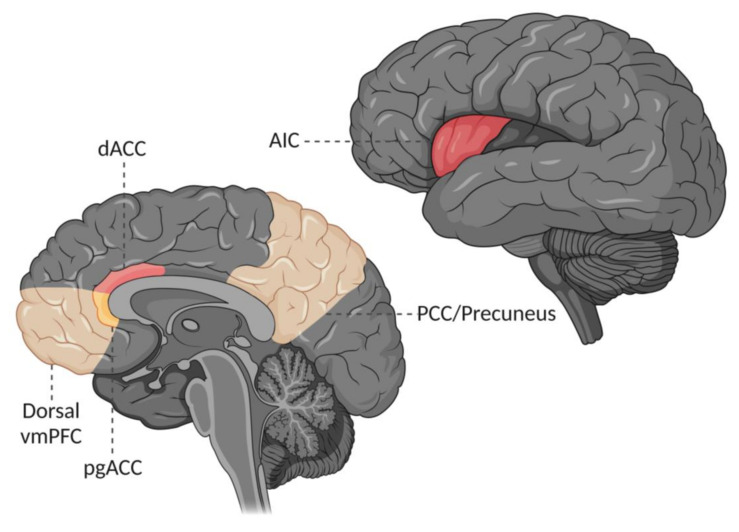
Core regions of the default mode network (orange) and salience network (pink). vmPFC = ventromedial prefrontal cortex; pgACC = pregenual anterior cingulate cortex; dACC = dorsal anterior cingulate cortex; PCC = posterior cingulate cortex; AIC = anterior insular cortex.

Acute pain plays an important role in warning us of actual or impending tissue harm [1]. However, when pain persists beyond the natural course of healing, it tends to lose many of its otherwise protective features, rather becoming a source of both physical and psychological suffering [2]. Many studies have tried to improve our understanding of chronic pain by exploring it from the perspective of the brain [3], and much progress has been made in the last decade concerning the understanding of how specific patterns of brain activity influence the pain experience [4]. One area of interest has been to interpret chronic pain through established, functional resting-state networks, with the salience network (SN) and default mode network (DMN) having gained much of the spotlight in previous chronic pain models [5,6,7,8,9,10]. The SN is centred around the anterior insular cortex (AIC) and the dorsal anterior cingulate cortex (ACC; Figure 1). It activates in response to personal salience [11,12] of both positive and negative valence [13,14,15,16]; that is, events that “stand out” in the personal environment [17], including acute pain [18,19], but it is also engaged during autonomic [20,21] and emotional regulation [14,22,23,24]. Furthermore, both the AIC and dorsal ACC are also included in the ventral attention network [25], which together with the dorsal attention network controls attentional relocation [26,27]. In contrast, the DMN was first discovered as a network of regions showing high metabolic activity during rest, whereby it became recognized as a network representative of the brain’s “default state” [28]. Today, activation of the DMN is known to reflect complex mentation processes [29,30], such as autobiographical memory retrieval and prospection [31,32,33], as well as self-generated spontaneous thought [34,35,36]. Its core regions include the posterior cingulate cortex extending to the precuneus, and the dorsal aspect of the ventromedial prefrontal cortex (vmPFC), with especially prominent intrinsic connectivity to the more posterior aspect of the dorsal vmPFC in and around the pregenual ACC (pgACC; Figure 1) [37], and in contrast to the SN, these regions normally exhibit significant deactivation in response to acute pain [19].

In people with chronic pain, meta-analyses have found no difference in acute experimental (stimulus-induced) pain-evoked DMN activity when compared to healthy, pain-free individuals, whereas inconsistent results have been reported for the SN [38,39,40]. However, many studies show increased functional connectivity between the AIC and the dorsal vmPFC [41,42,43,44,45,46,47,48], as well as altered functional connectivity between the dorsal vmPFC and the posterior DMN [41,44,45,46,49,50], in people with chronic pain when they experience spontaneous pain (i.e., pain experienced in the absence of external stimuli). Spontaneous pain has also been associated with altered oscillatory power frequencies within the dorsal vmPFC itself in people with chronic pain [41,42,50,51], and reduced deactivation of the dorsal vmPFC has been reported during both simple visuo-motor tasks [52,53,54] and attention-demanding cognitive tasks [55,56] in people with chronic pain when compared to healthy controls. We recently proposed a model in which we try to explain these functional changes in terms of an aberrant appraisal of threat in people with chronic pain [10]. We also highlight the possible importance of the vmPFC because of its frequent involvement in these changes. In this topical review, we therefore take a more regional standpoint, with a specific focus on altered activity in the dorsal vmPFC, as well as the altered functional connectivity between the dorsal vmPFC and the AIC observed in people with chronic pain. We aim to provide an updated model for what these cortical changes may represent, thereby hoping to extend the current perspective of the cortical mechanisms of chronic pain.

## 2. Possible Causes of Altered Dorsal Ventromedial Prefrontal Cortex Behaviour in People with Chronic Pain

### 2.1. Pain Versus No Pain during Scanning

There is an ongoing discussion about whether the observed differences in dorsal vmPFC activity and functional connectivity between people with chronic pain and healthy, pain-free individuals simply reflect the presence of pain itself [10,48,50]. Critically, blunted differences in functional connectivity between people with chronic pain and healthy controls have been observed when the presence of pain is experimentally controlled [48,50], and, in some studies, altered resting-state functional connectivity is only evident following pain exacerbation [45,46,47]. Furthermore, similar functional connectivity alterations to those observed in people with chronic pain, when they experience spontaneous pain in a resting state, have been observed in healthy individuals exposed to tonic experimental pain [50,57,58]. Although still to be confirmed, these results suggest that the altered behaviour of the dorsal vmPFC observed in people with chronic pain might be dependent on the presence of pain during scanning.

### 2.2. Inter-Individual Differences in Pain Processing

Most healthy, pain-free individuals display decreasing activity in the dorsal vmPFC during experimental pain [59], with the level of deactivation mediating subjective pain intensity [60]. However, a large-scale study of just over 400 healthy participants recently found that a substantial proportion (~36%) exhibits increased pain-evoked activity in the dorsal vmPFC in response to experimental, stimulus-induced pain [59]. Similarly, Mayr et al. recently showed that the same inter-individual variability in dorsal vmPFC activity, as well as functional connectivity, was present in people with chronic low back pain and migraine when they continuously rated their fluctuations in spontaneous pain intensity [61,62]. This may imply that an altered functional behaviour of the dorsal vmPFC may not reflect the state of chronic pain per se, but possibly inter-individual differences in pain processing. For instance, independent research showed that increased functional connectivity between the dorsal vmPFC and the nucleus accumbens predicted the transition from subacute to chronic low back pain [52,63,64], suggesting that the behaviour of the dorsal vmPFC may already at an early stage of pain increase the susceptibility of developing chronic symptoms in some individuals. Alternatively, the transition from (sub)acute to chronic pain may in itself be associated with increasing activity in the dorsal vmPFC [52]. The latter observation was reported in an early longitudinal study of people who transitioned from subacute to chronic low back pain [52]. However, as this study has not been replicated, the support for a shift in dorsal vmPFC activity during the transition to chronic pain is limited.

## 3. Revisiting Previous Models of Altered Dorsal Ventromedial Prefrontal Cortex Behaviour in People with Chronic Pain

In the following sections, we discuss the strengths and limitations of our own, as well as some of the most well-cited previous models which try to explain the altered dorsal vmPFC activity and functional connectivity in people with chronic pain. For more in-depth reading, we refer the reader to earlier publications specifically dedicated to each model [5,6,7,8,9,10].

### 3.1. Increased Emotional Processing of Pain

An early, well-recognized explanatory model for the altered behaviour of the dorsal vmPFC in people with chronic pain suggested a shift from mainly somatosensory, to mainly emotional cortical processing of pain [6,7]. One of the main functional domains of the dorsal vmPFC is indeed the regulation of emotion and affect [14,22,23,24,65,66]. Accordingly, human lesions to the vmPFC are associated with both apathy and blunted affect, as well as a lack of empathy [67]. Many of the cortical regions engaged during the experience of pain are also engaged during the expression of emotion [68], and experimentally induced negative emotion has been found to increase spinal reflexes (an index of spinal nociception) via increased activation of both the dorsal and ventral aspect of the vmPFC [69]. However, despite the established role of the dorsal vmPFC in emotional and affective regulation, its complete functional spectrum is highly multidimensional and also includes domains such as valence and reward processing [13,15,16,65,70,71], decision-making [65,72,73,74,75], memory retrieval and prospection [65,73], and self-referential processing [76,77,78]. Accordingly, in addition to emotional dysregulation, individuals with vmPFC lesions also exhibit behavioural changes characterized by increased impulsivity and irresponsibility [67], as well as an impaired or blunted ability to adapt their behaviour to previous experiences and environmental cues [67,79], including in contexts of pain [80]. Hence, increased emotional processing of pain may serve as a partial explanation for the altered behaviour of the dorsal vmPFC, but does it provide the complete explanation?

### 3.2. Aberrant Appraisal of Threat in the Context of Pain

The experience of pain is, by definition, “associated with, or resembling that associated with, potential tissue damage” [81]. Given the wide spectrum of functions of the dorsal vmPFC, another track of conceptual thought expressed across multiple models [8,9,10] has therefore been that chronic pain might resemble a more general misperception of pain and its contextual environment as potentially “dangerous”, even when the reality is “safe”. Similar to experimental pain, the experience of experimentally induced fear evokes similar deactivation of the dorsal vmPFC and activation of the SN [82]. As aforementioned, people with chronic pain are frequently found to show an impaired ability to deactivate the dorsal vmPFC during both simple and complex attention-demanding tasks while experiencing spontaneous pain [52,53,54,55,56]. However, in contrast to the heterogeneous responsiveness of the dorsal vmPFC observed in the context of pain outlined in previous sections, fear and threat learning are consistently associated with distinct deactivation of the dorsal vmPFC [83,84,85,86], whereas increased activation of this area is observed during fear and threat extinction [83,86,87,88]. Furthermore, although recent studies suggest that the vmPFC may not solely constitute a “safety hub” [79,89], such opposing activity responses are concentrated to the more ventral/posterior aspects of the vmPFC [89]. Accordingly, inactivation of the cortical equivalent to the dorsal vmPFC in non-human primates (i.e., area 32) increased behavioural fear responses during fear learning, while impairing fear extinction, whereas the opposite pattern was observed for inactivation of the more ventral/posterior aspect of the vmPFC (i.e., area 25) [90]. Thus, although it is tempting to suggest that chronic pain might be accompanied by a shift in the threat/safety profile of the dorsal vmPFC, is this plausible?

### 3.3. Increased Internal Attention to Pain

A third explanatory model for the altered behaviour of the dorsal vmPFC in people with chronic pain was presented by Kucyi and Davis, who suggested that it reflected increased internal attention to pain, which might disrupt the top-down endogenous pain modulatory pathway [5]. Specifically, Kucyi and Davis performed a series of experiments [91,92] in which they first observed that healthy individuals exposed to experimental, stimulus-induced pain exhibited an increased activation of the dorsal vmPFC, as well as an increased functional connectivity between the dorsal vmPFC and the periaqueductal gray (PAG) when they spontaneously attended away from pain (i.e., spontaneous non-pain-related mind wandering). Conversely, individuals who spontaneously directed their attention to the experimental pain exhibited reduced dorsal vmPFC activation, as well as an expected increased activation of the ventral attention network, including the core regions of the SN [92]. During a second experiment in people with chronic temporomandibular pain, an increased resting-state functional connectivity between the dorsal vmPFC and PAG was associated with pain rumination, as well as an increased functional connectivity between the dorsal vmPFC and the posterior cingulate cortex/precuneus [91]. Given the established role of the PAG in top-down endogenous pain modulation [93,94], the authors suggested that people with chronic pain who exhibit an altered functional behaviour of the dorsal vmPFC might be more likely to spontaneously ruminate about pain, which in turn may disrupt the communication between the dorsal vmPFC and the anti-nociceptive system [5]. However, given the results observed in the healthy participants [92], increased attention to pain would be expected to reduce the activity in the dorsal vmPFC, as well as the remaining regions of the DMN, possibly explaining the increased functional connectivity between the dorsal vmPFC and the posterior cingulate cortex/precuneus observed in the people with chronic temporomandibular pain [91]. In other words, it is contradictory to the increased activity in the dorsal vmPFC that is frequently reported in people with chronic pain during spontaneous pain [52,53,54,55,56].

## 4. An Updated Perspective on the Meaning of Altered Dorsal Ventromedial Prefrontal Cortex Behaviour in People with Chronic Pain

### 4.1. Shared Mechanisms with Placebo Analgesia

Recent findings suggest that a similar response heterogeneity of the dorsal vmPFC as that observed during acute experimental (i.e., stimulus-induced) [59] and chronic spontaneous pain [61] can also present during certain types of endogenous pain modulation. Contradictory to what might be expected given the frequently reported increased activity in the dorsal vmPFC in people with chronic pain [52,53,54,55,56], the vast majority of endogenous pain modulation trials in which healthy individuals exhibit increased activity in the dorsal vmPFC also report an associated reduction in subjective pain intensity and/or unpleasantness. This includes, for instance, relative pain relief (i.e., the pain relief experienced when a moderate-intensity stimulus is presented following a stimulus of high intensity) [95,96], positive reappraisal of pain [97,98], distraction analgesia [99,100], and when pain intensity is expected to be low [101,102] or reduced [103,104]. Increased activity in the dorsal vmPFC has also been found to encode pain-specific, positive reinforcement learning [105,106], and there is even an increased pain-evoked activation of the dorsal vmPFC if a painful stimulus has been accepted on behalf of one’s romantic partner [107].

There are, however, critical exceptions to this trend [102,108,109,110,111,112,113], with one of the main examples being the recent results of Zunhammer et al. concerning placebo analgesia [114]. In accordance with the common trend, early meta-analyses of placebo analgesia report increased pain-evoked activity in the dorsal vmPFC [103,104]. Yet, there are also well-designed studies which report the inverse pattern; that is, reduced activity associated with a greater placebo effect [109,110]. As recently raised by Zunhammer and colleagues, a major limitation of earlier meta-analyses is their reliance on published activation peaks rather than full activation maps, as this inherently increases the risk of biased results [115]. To get around this problem, the authors performed a meta-analysis of single-participant, whole-brain images across 20 independent studies, by which they intriguingly identified a great between-study heterogeneity in the response of the dorsal vmPFC [114]. This implies that a similar response heterogeneity of the dorsal vmPFC as that observed during acute experimental [59] and chronic spontaneous pain [61] is also present during placebo analgesia.

### 4.2. The Possible Role of the Pregenual Anterior Cingulate Cortex and Independence of Afferent Nociception

Importantly, placebo analgesia typically engages the pgACC subregion of the dorsal vmPFC [103,104], which not only resembles an important functional hub within both the DMN [37], but which also exhibits strong functional connections to the PAG [116]. Critically, the pgACC also resembles one of the areas most frequently reported to exhibit altered activity and/or functional connectivity in people with chronic pain [44,45,47,51,53,54,55,56,61,117]. Activity in the pgACC (among other regions) has been found to predict the experience of experimental pain independent of stimulus intensity [118], and data from patients with lesions to the vmPFC, including the pgACC, showed no difference in neither thermal pain threshold nor tolerance compared to healthy controls [80]. Thus, although nociceptive transmission is often facilitated in people with chronic pain via sensitization of peripheral and/or central nociceptive neurons [119,120,121], such afferent nociceptive mechanisms may not explain the altered functional behaviour of the pgACC in people with chronic pain.

Conversely, experimental pain-evoked activation of the pgACC has been more closely related to the expected value of pain rather than the factual stimulus-evoked pain experience itself [101,102,103,105,106,110,122]. Expectations are well-known to be able to shape the experience of pain [123], as well as other sensory perceptions [124], and expectations resemble one of the most well-established contributors to the placebo analgesic effect [123,125,126,127]. However, recent results by Atlas et al. showed that expectations of pain intensity were associated with different pain-evoked activity responses in the pgACC depending on how the cue that caused the expectations had been learned [102]. If the meaning of an auditory cue preceding a painful stimulus had been learned via uninstructed learning (i.e., learning by experience), expectations of high pain were associated with a significant pain-evoked deactivation of the pgACC. In contrast, if the meaning of the cues had been learned through explicit verbal instructions, expectations of high pain were rather associated with increased pain-evoked activation of the pgACC [102]. These results suggest that the altered behaviour of the pgACC observed in people with chronic pain may not be related to the expectations of pain per se. Yet, as instructed learning is a conscious way of learning [123], pgACC hyperactivity in people with chronic pain may reflect a conscious mentation process that covaries with expectations of pain.

### 4.3. Overthinking about the Meaning of Pain for Oneself and One’s Actions

**Figure 2:**
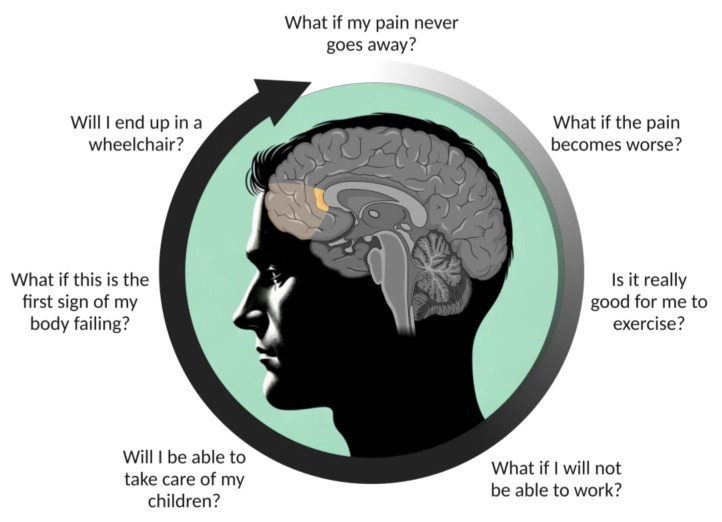
Examples of thoughts in people with chronic pain that might be associated with activation of the pregenual anterior cingulate cortex (bright orange) within the dorsal ventromedial prefrontal cortex (transparent orange).

Recently, Zhang and colleagues suggested that activity within the pgACC may encode the level of uncertainty related to pain [128]. The authors exposed healthy individuals to a tonic painful heat stimulus, during which they had to learn, via trial and error, which out of two buttons was associated with a short period of pain relief. Interestingly, the higher the uncertainty about which button to press, the higher the activity in the pgACC [128]. However, other studies found no involvement of the pgACC during uncertainty when there was no motor task performed [129,130,131], which suggests that the uncertainty component observed by the group of Zhang and colleagues most likely does not reflect uncertainty as to whether the tonic pain would be relieved or not, but to which choice to make to achieve pain relief. Similarly, a series of rigorous studies by Kolling et al. outside the context of pain have shown that the pgACC is activated during decision making but does not encode factual decision value [73,132]. In contrast, pgACC activity was suggested to reflect prospective consequential thinking, irrespective of the factual value of the participant’s final decision [73]. Together with the results by Zhang et al. [128], these findings suggest that activity in the pgACC might reflect the conscious weighing of the potential future consequences of one’s behavioural choices. However, repeated meta-analyses have found that the pgACC also increases in activity when individuals are actively reflecting or making judgements related to the self in general [76,77,78]. Altogether, this may imply that individuals with chronic pain who exhibit increased pain-evoked activity in the pgACC may be more likely to reflect on what their pain means for them and their actions (Figure 2). If negative, overthinking of such character may, at least in part, explain some of the common psychosocial characteristics of many people with chronic pain, such as trouble falling asleep and/or maintaining sleep, emotional distress (e.g., anxiety, depression), and/or avoidant behaviours (e.g., physical inactivity, social isolation) [2].

## 5. Anterior Insula Hyper-Connectivity to the Dorsal Ventromedial Prefrontal Cortex

No brain region works in isolation, and as mentioned in the introduction, one of the most frequently reported functional connectivity deviations in people with chronic pain is an increased functional connectivity between the dorsal vmPFC and the AIC [41,42,43,44,45,46,47,48]. In addition to its consistent activation in response to salience, the AIC has also been recognized as a region critical for interoception [11,12,133,134]; that is, the cortical processes underlying our ability to feel internal signals arising within our own body (e.g., nociception) [133,134]. In accordance, increasing AIC activity has been found to track increases in spontaneous pain intensity in people with chronic pain [53,61] and to predict subjective experimental pain intensity in healthy individuals [60,135]. Furthermore, activation of the AIC was recently found to mediate the relationship between actual stimulus intensity and subjective pain intensity [60].

However, similar to the dorsal vmPFC, activation of the AIC can also be modulated independent of stimulus intensity. For example, pain-evoked AIC activity was increased when contextual circumstances induced an increased subjective pain intensity or unpleasantness, such as when expectations of high or reduced pain were inferred [101,102,103,122,136], when a painful stimulus of moderate intensity was presented following a low-intensity stimulus [95], or when participants engaged in cognitive downregulation of pain [97]. Furthermore, AIC activity can also increase during the anticipation of pain [137], and if a given stimulus is more painful than expected, activation of the AIC has been found to increase further both during and anticipatory to the subsequent stimulus [105,106]. These observations suggest that activity in the AIC might be related to the perceived intensity of pain. However, given the prominent effect of contextual modulation on the level of AIC activity, this relationship may not rely solely on afferent nociception. In the context of pain, AIC activity may thus possibly reflect a more general type of pain-related processing, such as the level of personal “threat” of a given context [9,10].

**Figure 3:**
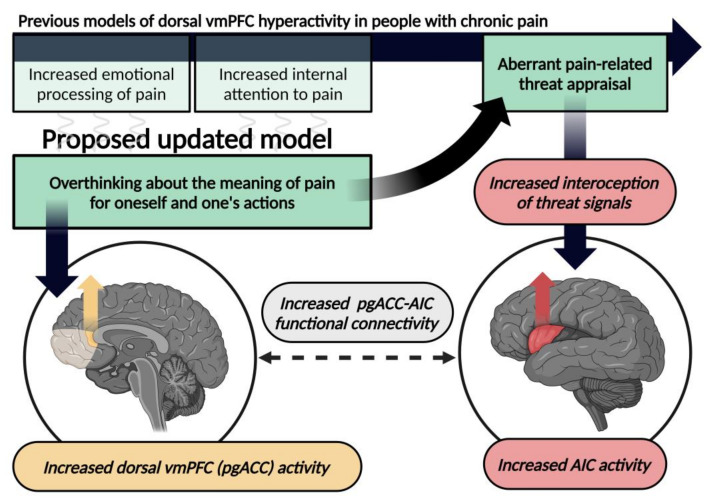
A proposed updated model for the meaning of the altered behaviour of the pregenual anterior cingulate cortex (pgACC), within the dorsal ventromedial prefrontal cortex (vmPFC), in people with chronic pain. Overthinking about the meaning of pain is suggested to be associated with increased activation of the pgACC within the dorsal vmPFC. This cognitive behaviour may subsequently lead to an aberrant appraisal of threat in the context of pain via increased interoception of threat signals (e.g., nociception) processed in the anterior insular cortex (AIC). The simultaneous increased activation in the pgACC and AIC may present as an increased functional connectivity between the two cortical regions.

If our previously suggested role of the pgACC in (chronic) pain processing holds true, increased functional connectivity between the pgACC and AIC, as often observed in people with chronic pain [41,42,43,44,45,46,47,48], may thus possibly reflect a process by which negative overthinking about the meaning of pain for oneself and one’s actions might increase the personal “threat” of a given context. This may in turn increase the likelihood of “harmless” afferent signals from the body to be interpreted as “potentially harmful”, thereby increasing the susceptibility of experiencing pain in the absence of ongoing tissue damage (Figure 3).

## 6. Conclusions

In the present review, we discuss the altered functional behaviour within DMN and SN in people with chronic pain, with a special focus on the dorsal vmPFC and its functional connections to the AIC. By integrating previous theoretical models with novel research findings, we suggest an updated model of what both the altered activity in and functional connectivity to the dorsal vmPFC may represent in people with chronic pain. We suggest that increased dorsal vmPFC activity may reflect a tendency to overthink the meaning of pain for oneself and one’s actions. This may in turn increase the personal threat of a given context and thereby increase the susceptibility to experience pain, which we suggest might be reflected by an increased functional connectivity between the dorsal vmPFC and the AIC.

## Data Availability

Not applicable.
